# Dendrimer-Derived Mimics of Host Defense Peptides Selectively Disrupt Cancer Cell Membranes for Melanoma Therapy

**DOI:** 10.3390/pharmaceutics17030361

**Published:** 2025-03-12

**Authors:** Yusheng Qian, Danjing Yang, Xiangyu Lin, Chenyun Shen, Jieping Zhang, Jin Xu, Yan Zhao, Ling Zhu, Haoran Kong, Mingyu Zhang, Yueqian Zhu, Chuncai Zhou, Jing He

**Affiliations:** 1 Translational Medical Center for Stem Cell Therapy, Department of Dermatology, Tongji Hospital, School of Medicine, Tongji University, Shanghai 200331, China; 2011233@tongji.edu.cn (Y.Q.); djyang@tongji.edu.cn (D.Y.); linxiangyu668@gmail.com (X.L.); 2050460@tongji.edu.cn (C.S.); zhangjieping@tongji.edu.cn (J.Z.); 2150816@tongji.edu.cn (H.K.); 2153107@tongji.edu.cn (M.Z.); 2 School of Material Science and Engineering, Tongji University, 4800 Caoan Road, Shanghai 201804, China; 3 Laboratory Animal Center of Tongji University, Tongji University, Shanghai 200092, China; sydwzx@tongji.edu.cn; 4 Experimental Teaching Center for Medicine and Life Science, School of Medicine, Tongji University, Shanghai 200331, China; 90801@tongji.edu.cn (Y.Z.); yxyzhuling@mail.tongji.edu.cn (L.Z.); 5 Department of Dermatology, The First Affiliated Hospital of Soochow University, Suzhou 215031, China; zhuyueqian@suda.edu.cn

**Keywords:** host defense peptides, melanoma, anticancer polymers, membranolytic mechanism

## Abstract

**Background**: Melanoma is one of the most common malignancies, posing a significant health threat to patients, particularly in advanced stages due to its high aggressiveness. Chemotherapy agents with biocompatibility and low susceptibility to induce resistance are required for systematic management. **Methods**: Dendrimer-derived mimics (DMs) of host defense peptides (HDPs), which were constructed by a dendrimer core and optimized ratios of the hydrophobic arm, were used to treat A375 cells and HaCaT cells as the control. Live/dead staining, flow cytometry, and scanning electron microscopy (SEM) were conducted to analyze the anticancer mechanism. Mice with subcutaneous tumors were used to test the antitumor activity and toxicity in vivo. **Results**: DMs exhibited enhanced activity against A375 cells with remarkable selectivity, which mimics the action of natural HDPs and can cause damage to cell membranes. DMs can effectively inhibit solid tumor growth with minimal systemic toxicity and no adverse effects on healthy tissues. **Conclusion**: All the findings highlight DMs as promising anticancer candidates with significant potential for systemic melanoma therapy.

## 1. Introduction

Melanoma is a highly aggressive skin cancer with a rising global incidence, making it a significant clinical challenge [1,2,3]. Advanced-stage melanoma is particularly concerning, because its metastasis drastically reduces patients’ median survival rates [4,5,6]. Despite advancements in immunotherapy that have mitigated melanoma-related mortality, systematic management remains inadequate for refractory and relapsed cases due to the lack of effective chemotherapy options [7,8,9]. This limitation largely stems from the unfavorable profiles of traditional chemotherapeutic agents, such as dacarbazine, which demonstrate high systemic toxicity, the possibility to induce drug resistance, and poor tumor site accumulation [10,11]. Addressing these shortcomings is crucial for the development of novel therapeutic agents to combat melanoma more effectively.

Host defense peptides (HDPs) have emerged as promising anticancer candidates due to their unique membranolytic mechanism against bacteria and even cancer cells [12,13,14]. Unlike normal cells, cancer cell membranes display an excess of negative charges which were attributed to the abnormal externalization of phosphatidylserine on the outer leaflet [15,16]. This distinct feature enables cationic HDPs to selectively bind to cancer cells via electrostatic interactions, subsequently disrupting the cell membrane and inducing lysis [16]. This mechanism not only provides HDPs with potent anticancer activity but also reduces the possibility of developing resistance compared to traditional chemotherapy agents [16]. However, these advantages are counterbalanced by significant challenges, including low productivity, systemic toxicity, and poor enzymatic stability, which severely limit their clinical translation [17,18].

Cationic amphiphilic polymers have been recently designed to mimic the membranolytic mechanism of HDPs while addressing their inherent limitations [19,20]. Among all the biomaterials, dendrimers as three-dimensional, highly branched polymers stand out as superior biomaterials due to their unique structural and functional advantages [21]. Compared to linear polymers, dendrimers offer an extended in vivo half-life, enhanced binding affinity to cell membranes, and potential immunomodulatory effects, making them particularly well-suited candidates for biomedical applications [22,23]. However, reports on the dendrimer-based HDP mimics remain scarce, and their effect against different cancer cells has yet to be comprehensively explored.

**Figure 1:**
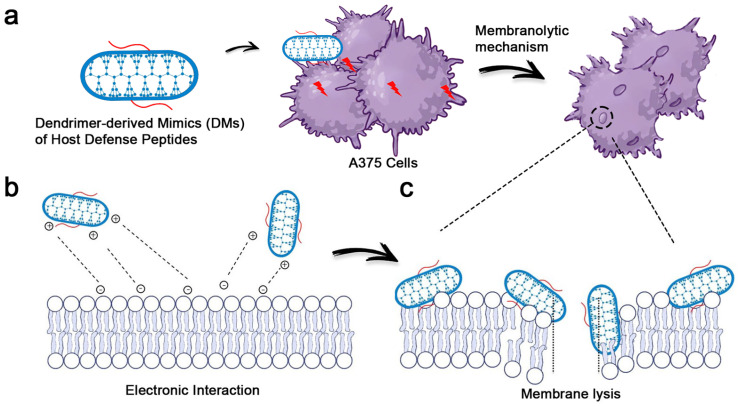
Scheme of the membranolytic mechanism of DMs against A375 cells. (**a**) Schematic representation of DMs. DMs destroy the membrane of A375 cells by inducing pore formation and membrane contraction. (**b**) DMs with cationic charges absorb the cell membrane via electronic interaction as the cancer cell membrane possesses negative charges. (**c**) DMs further insert into the cell membrane, causing pores as a consequence.

In our previous work [24], we developed dendrimer-derived mimics (DMs) of HDPs, featuring a hydrophilic dendronized polylysine core and hydrophobic polycaprolactone (PCL) arms. These DMs demonstrated significant potential in treating cancer, and we hypothesized that they could achieve superior efficacy in treating superficial tumors, such as skin cancer. Herein, we systematically evaluated the anticancer activity of dendronized polylysine with varying generations and DMs against melanoma A375 cells. To assess their selectivity, cytotoxicity against HaCaT cells was also examined. This investigation elucidated the relationship between dendrimer structure and its anticancer activity. Mechanistic studies, including scanning electron microscopy (SEM), live/dead staining, and flow cytometry, revealed the unique membranolytic action of our DMs. Furthermore, in vivo anticancer efficacy was assessed using subcutaneous melanoma models in nude mice (Figure 1). Our DMs demonstrated potent anticancer activity and remarkable selectivity, highlighting their potential as promising candidates for melanoma treatment and underscoring the utility of dendrimer-based structures as HDP mimics.

## 2. Materials and Methods

### 2.1. Materials

The dendronized polylysine cores (D_G1_, D_G2_, and D_G3_), PCL, and a series of D_G3_M_n_ (*n* = 2, 4, 6, 8, where n represents the number of PCL arms modified on D_G3_, Figure S1 and Figure 1a) were synthesized following previously reported methods [24]. Briefly, the dendronized polylysine cores were prepared with stepwise conjugation of activated lysine to the preceding generation. Subsequently, PCL arms functionalized with isocyanate groups were obtained via ring-opening polymerization and were conjugated to D_G3_ through efficient reactions with amino groups, yielding the D_G3_M_n_ series. For subsequent experiments, the dendrimer-derived mimics (DMs) were prepared by dissolving 10 mg of each DM in 1 mL of phosphate-buffered saline (PBS; 1×, Corning, Corning, NY, USA). The prepared solutions were filtered using a 0.22 µm sterile filter (Taitan, Shanghai, China) to ensure sterility and uniformity before use.

### 2.2. Cell Culture

The human malignant melanoma A375 cell line and normal human keratinocyte HaCaT cell line were obtained from the National Collection of Authenticated Cell Cultures (Shanghai, China). A375 cells and HaCaT cells were cultured in Dulbecco’s modified eagle medium (DMEM; Gibco, Carlsbad, CA, USA) supplemented with 10% fetal bovine serum (FBS; Gibco, Carlsbad, CA, USA) and 1% penicillin/streptomycin (P/S; Gibco, Carlsbad, CA, USA). Cells were maintained in an incubator at 37 °C with 5% CO₂. The culture medium was refreshed every 48 h to sustain optimal cell growth. For subculturing, cells were detached using 0.05% trypsin-EDTA solution (Gibco, Carlsbad, CA, USA) when reaching approximately 80% confluence, with a split ratio of 1:3. Only low-passage cells (passages 2–6) of HaCaT and A375 were used in all subsequent experiments to ensure reproducibility and minimize phenotypic drift.

### 2.3. CCK-8 Analysis

The cytotoxicity of DMs was evaluated using the cell counting kit-8 (CCK-8, Dojindo, Kumamoto, Japan) assay. A375 cells and HaCaT cells were seeded into 96-well plates at a density of about 5 × 10^3^ cells/well in 100 µL of culture medium, with five replicates for each experimental group. After an initial incubation for 24 h to allow cell attachment, the DMs were diluted in the respective culture medium to final concentrations of 0, 8, 16, 32, 64, 128, and 256 μg/mL. The cells were treated with the DMs at these concentrations for 6, 24, 48, and 72 h. At each time point, 10 µL of CCK-8 solution was added to each well and incubated at 37 °C for an additional 2.5 h. The absorbance at 450 nm was measured using a microplate reader (Thermo Scientific Varioskan Flash, Waltham, MA, USA). Untreated cells served as the negative control, and the medium without cells was used as the blank control. The cell viability was calculated as a percentage of cell viability compared to the control group.

### 2.4. Live/Dead Staining Analysis

Live/dead staining was performed to evaluate the effects of D_G3_M_2_ (A dendronized polylysine cores D_G3_ with two modified PCL arms) on cell viability and its potential to cause membrane damage. A375 cells and HaCaT cells (5 × 10^4^ cells/well) were seeded into 24-well plates and allowed to attach for 24 h before treatment. For dose-dependent analysis, cells were treated with D_G3_M_2_ at final concentrations of 0, 8, 16, 32, 64, 128, and 256 μg/mL for 12 h. For time-dependent analysis, cells were treated with 64 μg/mL D_G3_M_2_ for various time intervals (30, 60, 120, 180, and 240 min). After treatment, the cells were stained with a live/dead viability kit (Calcein AM/PI; KeyGen Biotech, Nanjing, China). Fluorescent images of live (Calcein AM, green fluorescence) and dead (PI, red fluorescence) cells were captured using an inverted phase-contrast fluorescence microscope (Nikon Eclipse Ti-S, Tokyo, Japan).

### 2.5. Flow Cytometry Analysis of Apoptosis

To evaluate cell apoptosis, 5 × 10^5^ A375 cells were seeded into 6-well plates and cultured for 24 h to allow attachment. D_G3_M_2_ at various concentrations (0, 8, 16, 32, 64, and 128 μg/mL) was added to the wells and incubated with the cells for 12 h. After treatment, the cells were collected and washed twice with cold phosphate-buffered saline. Cell apoptosis was assessed using the Annexin V-FITC/PI Apoptosis Detection Kit (Yeasen, Shanghai, China). Briefly, cells were incubated with 5 µL of annexin V-FITC and 5 µL of propidium iodide (PI) for 15 min at room temperature in the dark. Fluorescence signals were analyzed using a fluorescence-activated cell sorter (FACS; BD FACSAria III, BD Biosciences, San Jose, CA, USA) to distinguish live, early apoptotic, and late apoptotic/necrotic cells.

### 2.6. Scanning Electron Microscopy (SEM) Analysis

To evaluate the effects of DMs on cancer cell membranes, A375 cells (5 × 10⁴ cells/well) were seeded onto sterilized glass coverslips in 24-well plates and cultured for 24 h to allow attachment. The cells were then treated with 64 μg/mL DMs for 12 h. After treatment, the cells were fixed with 4% paraformaldehyde (PFA; Sigma-Aldrich, St. Louis, MO, USA) at room temperature for 20 min and washed three times with PBS to remove residual fixative. Gradient ethanol dehydration was performed sequentially with 70%, 80%, 95%, and 100% ethanol, each for 15 min. Dehydrated samples were dried and sputter-coated with a layer of gold to enhance conductivity. The samples were then examined using a scanning electron microscope (SEM, Zeiss, Berlin, Germany) to observe morphological changes in the cell membrane.

### 2.7. In Vivo Anticancer Analysis

A total of six female BALB/c nude mice (6 weeks old, body weight 18–20 g) were purchased from the Experimental Animal Center at Tongji University (Shanghai, China). All animal studies were conducted following the protocols approved by the Animal Experimental Ethical Inspection Committee of the Laboratory Animal Centre, Tongji University (Approval No. TJAA00422103). To establish subcutaneous melanoma models, 5 × 10^6^ A375 cells in 100 µL PBS were injected subcutaneously into the right flank of each mouse. Tumor dimensions were measured using a vernier caliper, with the tumor volume calculated using the following formula:

$$\mathrm{Tumor Volume}=\frac{1}{2}\times (\mathrm{long axis})\times {\left(\mathrm{short axis}\right)}^{2}$$

Once the average tumor volume reached approximately 30 mm^3^, the mice were randomly divided into two groups (*n* = 3 per group). One group received intraperitoneal injections of DMs (10 mg/kg in PBS), while the control group received an equivalent volume of PBS. Injections were administered every two days for a total of 28 days. Body weight and tumor dimensions were recorded before each injection to monitor systemic toxicity and tumor progression. At the end of the treatment, mice were euthanized humanely, and tumors were excised and weighed. Tissues were fixed in 4% paraformaldehyde, embedded in paraffin, sectioned, and stained with hematoxylin and eosin (H&E) for histopathological evaluation to assess systemic toxicity.

### 2.8. Statistical Analysis

Statistical analyses were performed using SPSS 25.0 (IBM Corporation, Armonk, NY, USA) and OriginPro (OriginLab Corporation, Northampton, MA, USA). Quantitative data are presented as mean ± standard deviation (SD) and were derived from at least three independent experiments. Comparisons between two groups were conducted using the Student’s *t*-test, while multiple group comparisons were analyzed by one-way analysis of variance (ANOVA) followed by post hoc tests when applicable. Statistical significance was set at *p* < 0.05. In figures, levels of significance are denoted as follows: *p* < 0.05 (*), *p* < 0.01 (**), and *p* < 0.001 (***).

## 3. Results

### 3.1. Potent Anticancer Activity and Remarkable Selectivity of DMs

**Figure 2:**
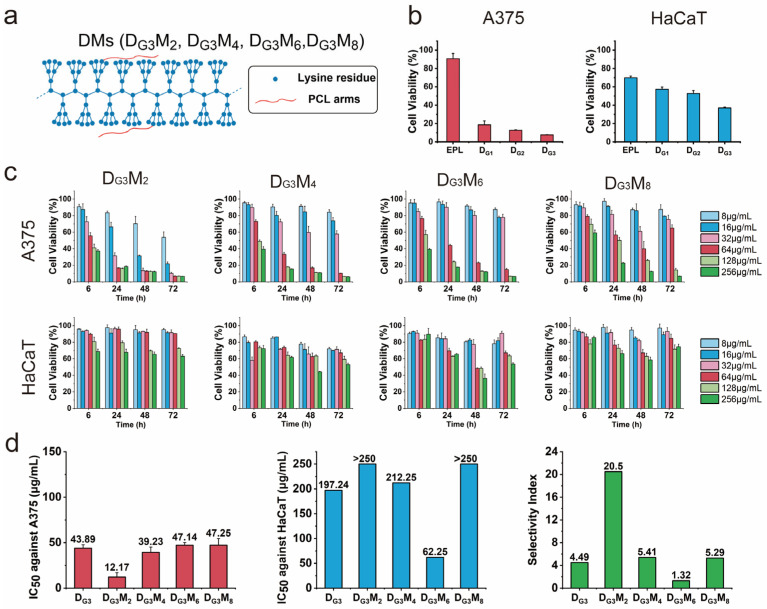
Cytotoxicity of dendronized polylysine core and DMs against A375 and HaCaT cells as determined by CCK-8 assay. (**a**) Schematic representation of DMs constructed by dendronized polylysine core and varying numbers of PCL arms. (**b**) Cell viability of A375 and HaCaT cells following treatment with dendronized polylysine core (256 μg/mL) for 72 h. (**c**) Cell viability of A375 and HaCaT cells treated with DMs at various concentrations and incubation times. (**d**) IC_50_ values of DMs against A375 and HaCaT cells and the selectivity index, calculated as the ratio of IC_50_ against HaCaT cells to IC_50_ against A375 cells.

To evaluate the relationship between cytotoxicity and dendrimer structure, the viability of A375 cells and HaCaT cells was assessed using the CCK-8 assay after treatment with dendronized polylysine core of varying generations (D_G1_, D_G2_, and D_G3_). As shown in Figure 2b and Figure S2, *ε*-polylysine, used as the backbone for dendronized polypeptide preparation, exhibited minimal toxicity toward A375 cells, with cell viability remaining at 90.6% ± 5.68%, even at the high concentration tested (256 μg/mL, 72 h). In contrast, dendronized polylysine cores (D_G1_, D_G2_, and D_G3_) showed increased cytotoxicity with higher generations. The viabilities of A375 cells decreased to 18.6% ± 4.23%, 12.6% ± 0.61%, and 7.5% ± 0.26%, respectively. A similar trend was observed in HaCaT cells, where viabilities decreased to 57.3% ± 2.43%, 52.7% ± 3.16%, and 37.0% ± 0.84%, respectively.

Based on the strongest cytotoxicity observed for D_G3_, dendronized polylysine mimics (DMs) were constructed starting from D_G3_. However, due to D_G3_’s low selectivity, PCL with excellent biocompatibility was incorporated into the structure. The resulting products, named D_G3_M_n_ (where *n* = 2, 4, 6, 8), were tested for cytotoxicity against A375 and HaCaT cells (Figure 2c). DMs displayed sustained capabilities to inhibit cancer cell growth over time, as the viability of A375 cells continued to decrease during the 72 h incubation period (Figure S3). Moreover, The half-maximal inhibitory concentrations (IC_50_) after a 48 h incubation were calculated using linear interpolation. As shown in Figure 2d, D_G3_M_2_, D_G3_M_4_, D_G3_M_6_, and D_G3_M_8_ exhibited IC_50_ values of 12.17 ± 4.74, 39.23 ± 5.93, 47.14 ± 2.97, and 47.25 ± 7.21 μg/mL against A375 cells, respectively. In contrast, DMs demonstrated significantly lower cytotoxicity against HaCaT cells. Specifically, D_G3_M_2_ and D_G3_M_8_ had IC_50_ values were greater than 256 μg/mL, while D_G3_M_4_ and D_G3_M_6_ exhibited relatively higher cytotoxicity, with IC_50_ values of 212.25 ± 5.94 and 62.25 ± 1.32 μg/mL, respectively. Specifically, selectivity indexes for each DM were calculated as the ratio of IC_50_ values against HaCaT cells to IC_50_ values against A375 cells. D_G3_M_2_ showed the highest selectivity (20.5), while the other products, including D_G3_, showed lower selectivity (below 5.41).

### 3.2. Demonstration of the Membranolytic Mechanism via the Live/Dead Staining Analysis

**Figure 3:**
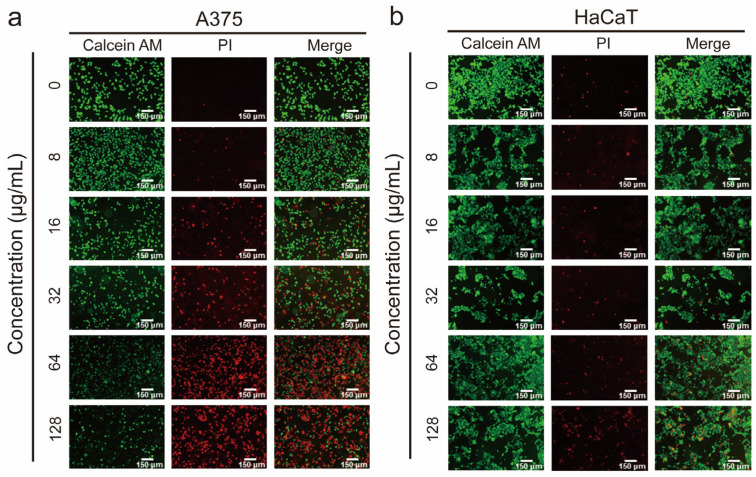
Concentration-dependent live/dead staining analysis of DMs against A375 (**a**) and HaCaT cells (**b**). Cells were treated with DMs at concentrations of 0, 8, 16, 32, 64, and 128 μg/mL for 6 h.

The outstanding performance of DMs against malignant cells in vitro motivated us to investigate whether their anticancer mechanism successfully mimics that of natural HDPs. Calcein AM was used to stain viable cells, while PI labeled the DNA of dead cells with disrupted membranes. Both concentration-dependent and time-dependent live/dead staining assays were performed. As shown in Figure 3a, the ratio of dead A375 cells increased with rising concentrations of D_G3_M_2_, ranging from 8 μg/mL to 128 μg/mL. Nearly 85% of A375 cells were dead after 12 h of treatment at the highest concentration. In contrast, while a similar concentration-dependent trend was observed, less than 30% of HaCaT cells were killed under the same conditions (Figure 3b). These findings align closely with the CCK-8 results, reaffirming the selective cytotoxicity of D_G3_M_2_ toward cancer cells. As for the killing kinetics assay, approximately 10% of A375 cells died within the initial 30 min of D_G3_M_2_ treatment, with complete eradication of viable cells achieved within 240 min (Figure S4). In contrast, HaCaT cells were minimally affected during this period, with less than 10% cell death observed even after 240 min. These results emphasized that D_G3_M_2_ showed a preference for disrupting cancer cell membranes.

### 3.3. The Membranolytic Mechanism Indicated by Flow Cytometry Analysis

**Figure 4:**
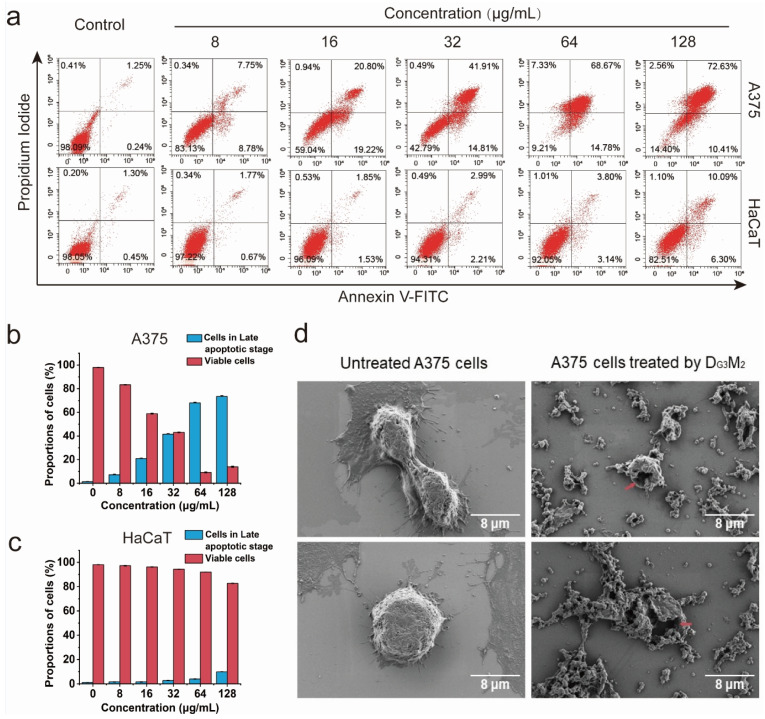
Flow cytometry analysis (annexin V/PI) of A375 and HaCaT cells after treatment with D_G3_M_2_ at various concentrations for 6 h. (**a**) Flow cytometry plots of A375 and HaCaT cells after treatment with D_G3_M_2_. (**b**) Proportion of late apoptotic and viable A375 cells after the treatment at different concentrations. (**c**) Proportion of late apoptotic and viable HaCaT cells after the treatment at different concentrations. (**d**) Morphological changes of A375 cancer cells before and after treatment with D_G3_M_2_. Treatment with D_G3_M_2_ significantly compromised cell membrane integrity (red arrows), resulting in observable morphological alterations.

Flow cytometry with annexinV-FITC/PI double staining was utilized to distinguish viable, early apoptotic, late apoptotic, and necrotic cells following D_G3_M_2_ treatment (Figure 4a). As shown in Figure 4b, the results demonstrated a significant reduction in the proportion of viable A375 cells, decreasing from 83.4% ± 0.27% to 14.0% ± 0.55% as the D_G3_M_2_ concentration increased from 8 μg/mL to 128 μg/mL. Most affected cancer cells were classified as late apoptotic, with their proportion rising from 7.23% ± 0.46% to 73.5% ± 0.85%. In contrast, early apoptotic and necrotic cells consistently remained at low levels across all concentrations. For HaCaT cells (Figure 4c), the proportion of viable cells remained as high as 82.51%, even at the maximum concentration of 128 μg/mL, supporting the observed selectivity of D_G3_M_2_.

### 3.4. Clear Membrane Lysis Observed by SEM Analysis

To gain deeper insights into the membranolytic mechanism, the optimized product D_G3_M_2_ was used to treat melanoma cells, and the resulting membrane alterations were observed using SEM. After 12 h of incubation, significant morphological changes were evident in A375 cells treated with D_G3_M_2_ (Figure 4d). Untreated A375 cells adhered firmly to the plate, exhibiting an oval shape with intact cell membranes and abundant filopodia. In stark contrast, cells treated with D_G3_M_2_ displayed severe membrane damage, including the formation of large holes in the cell membrane, through which intracellular contents visibly leaked out. Additionally, D_G3_M_2_ caused noticeable shrinkage of the filopodia, suggesting its potential to impair tumor cell invasiveness. Furthermore, numerous small vesicles were observed on the plate surface, likely remnants shed from the disrupted cell membranes. These findings provide compelling evidence that D_G3_M_2_ exerts its anticancer effects via a membranolytic mechanism, effectively mimicking the natural action of HDPs as our intended design.

### 3.5. The Capability of DMs to Inhibit Tumor Growth in Vivo with Low Tissue Toxicity

**Figure 5:**
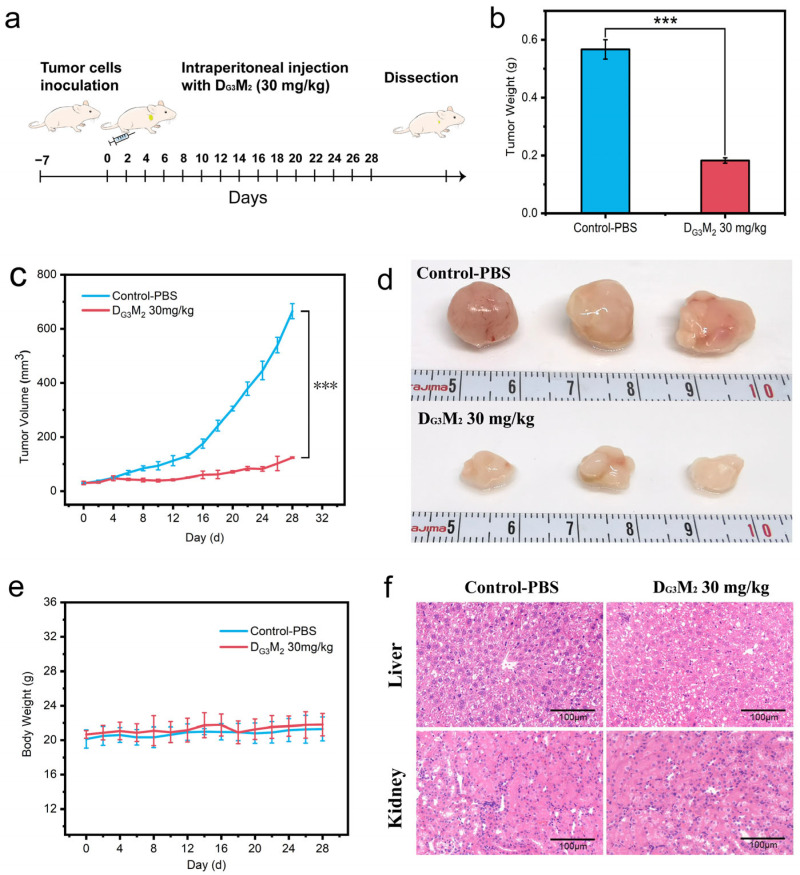
In vivo anticancer analysis of D_G3_M_2_ against A375 tumors. (**a**) Construction of mice model and the treatment of D_G3_M_2_ (30 mg/kg) (**b**) Tumor weight measured after the completion of the experiment, *p* < 0.001 (***). (**c**) Average tumor volume in different treatment groups throughout the experiment, *p* < 0.001 (***). (**d**) Photographs of dissected tumor tissues from mice after 28 days of treatment. (**e**) Body weight changes of mice during the experimental period. (**f**) H&E staining of liver and kidney tissues to assess potential organ toxicity.

The in vivo anticancer efficacy and safety profile of D_G3_M_2_ were evaluated using a subcutaneous tumor model in nude mice. A375 cells were injected into the mice to establish tumors, and treatments commenced when tumor volumes reached approximately 30 mm^3^. Mice were randomly assigned to two groups (*n* = 3 per group) and treated with D_G3_M_2_ or PBS via intraperitoneal injection for 28 days (Figure 5a). Tumor growth in the PBS-treated control group progressed rapidly, with tumor volumes exceeding 665 mm^3^ (Figure 5c), whereas the D_G3_M_2_-treated group exhibited significantly reduced tumor growth, maintaining volumes below 125 mm^3^ throughout the experiment. At the end of the study, tumor weights were measured, revealing a 68% reduction in the D_G3_M_2_ group (0.183 g, Figure 5b) compared to the control group. Mice body weights remained stable in both groups, indicating no apparent systemic toxicity associated with D_G3_M_2_ treatment (Figure 5e). Furthermore, hematoxylin and eosin (H&E) staining of liver and kidney tissues showed no histological abnormalities after 28 days of treatment (Figure 5f), further confirming the biocompatibility of D_G3_M_2_.

## 4. Discussion

Traditional chemotherapy often falls short in providing systematic treatment of skin cancer [25,26], underscoring the urgent need to develop novel anticancer agents with enhanced selectivity and biocompatibility. In our previous work [24], dendronized polylysine mimics (DMs) of natural HDPs demonstrated activity against bacterial pathogens and A549 lung cancer cells. However, their efficacy required further optimization. In this study, we explored the potential of DMs as selective and effective therapeutic agents for melanoma, offering a promising approach to address this unmet clinical need.

Our findings demonstrate a clear correlation between the generation of the dendronized polylysine core and its cytotoxicity. Among D_G1_, D_G2_, and D_G3_, the latter exhibited the strongest anticancer activity, which can be attributed to the increase in terminal amino groups. This trend aligns with observations for other cationic polymers, such as polyethyleneimine (PEI), where higher amino group density enhances cationic charge [27,28]. The increased charge density facilitates stronger interactions with negatively charged cancer cell membranes, driving the observed cytotoxicity. However, D_G3_ showed poor selectivity, significantly affecting both A375 melanoma cells and HaCaT keratinocytes which have become the key limitation for designing anticancer agents. To address this, PCL arms were incorporated to improve the selectivity of the dendronized polylysine core, and DMs were constructed.

For the anticancer assay, D_G3_M_2_ demonstrated the most potent activity against A375 cells, with the lowest IC_50_ (12.17 μg/mL). As the ratio of PCL increased, anticancer activity gradually decreased. This observation suggests that a low proportion of hydrophobic residues optimally enhances interactions between DMs and the lipid components of cancer cell membranes. However, excessive PCL incorporation fails to further improve these interactions and instead limits the solubility of the resulting polymer. Consequently, D_G3_M_8_ exhibited the weakest anticancer activity among the tested derivatives. Regarding cytotoxicity against HaCaT cells, D_G3_M_2_ showed a marked improvement in biocompatibility compared to D_G3_, attributable to the intrinsic biocompatibility of PCL. In addition, D_G3_M_2_ also performed better compared to chemotherapy drugs [29] and natural HDPs [30,31]. However, further increases in hydrophobic residues appeared to compromise biocompatibility, likely due to their enhanced interactions with normal cell membranes. Overall, the PCL arms demonstrated both positive and negative effects; they improved biocompatibility but also disrupted cell membranes and adversely affected water solubility. These complex interactions led to variations in selectivity, with D_G3_M_2_ exhibiting superior selectivity (20.5) compared to the core D_G3_ and other DMs. This structure–activity relationship highlights the importance of balancing hydrophobic and hydrophilic components to optimize the HDP mimics. Additionally, the anticancer activity of D_G3_M_2_ against A375 cells was higher than its activity against A549 cells, as previously reported [24]. This finding underscores the potential of DMs as promising agents for skin cancer treatment.

The anticancer mechanism of DMs was elucidated through live/dead staining, SEM analysis, and flow cytometry assays. Live/dead staining demonstrated that D_G3_M_2_ induced cancer cell death in a concentration- and time-dependent manner while sparing normal cells, consistent with the CCK-8 assay results. The red fluorescence observed with PI staining indicated membrane damage in cancer cells, as PI is impermeable to intact membranes [32]. SEM analysis provided direct morphological evidence of membranolytic activity, revealing large pores, leakage of cellular contents, and disruption of filopodia in D_G3_M_2_-treated A375 cells. Compared to the membrane changes observed in A549 cells in our previous study [24], a larger scale of membrane lysis was evident in A375 cells, likely attributable to differences in membrane composition. This suggests that A375 cells are more susceptible to disruption by HDP mimics, further supporting their potential as systemic agents in skin cancer treatment. Flow cytometry further confirmed that D_G3_M_2_ predominantly induced late apoptosis, with minimal involvement of early apoptotic or necrotic pathways. Membrane disruption facilitated PI penetration and labeling of intracellular components, allowing clear identification of late apoptotic cells [33]. Collectively, these findings demonstrate that D_G3_M_2_ exerts its anticancer effects through a unique membranolytic mechanism, aligning with its design to mimic the anticancer processes of natural HDPs.

The potent in vitro anticancer activity of D_G3_M_2_ translated effectively to an in vivo model. D_G3_M_2_ significantly inhibited tumor growth in A375-bearing nude mice without causing systemic toxicity, as evidenced by stable body weight and unaltered liver and kidney histology. Tumor volumes in the D_G3_M_2_ group were consistently suppressed, with a 68% reduction in tumor weight compared to the PBS control group. These results highlight the favorable pharmacokinetics, stability, and biocompatibility of D_G3_M_2_, which are critical attributes for clinical translation [34].

Moreover, D_G3_M_2_’s ability to suppress tumor growth while sparing healthy tissues suggests an optimal balance between efficacy and safety, which is often a significant challenge in cancer therapy. These results also underline D_G3_M_2_’s favorable pharmacokinetics and stability in vivo, potentially attributable to its dendronized structure and the incorporation of biocompatible PCL arms. The enhanced selectivity observed in vitro was consistently reflected in vivo, reinforcing its suitability for targeted melanoma treatment. We believe that the application of DMs in a preoperative context to systematically manage the tumor mass before surgical procedures may offer a strategic advantage in optimizing therapeutic efficacy and patient outcomes. Overall, these findings position D_G3_M_2_ as a promising candidate for further preclinical and clinical evaluations aimed at developing effective and safe therapies for melanoma.

## 5. Conclusions

Melanoma treatment requires not only effective chemotherapy but also agents that can minimize resistance development and exhibit low systemic toxicity. In this study, we explored dendrimer-derived mimics (DMs) of HDPs, which demonstrated potent anticancer activity against A375 melanoma cells. The dendronized polylysine core was shown to exhibit generation-dependent cytotoxicity, highlighting the critical role of dendrimer generation in enhancing anticancer effects. After modification with PCL arms, the biocompatibility of DMs was significantly improved, leading to increased selectivity for cancer cells over normal cells. Among the various derivatives, D_G3_M_2_ emerged as the most promising candidate, demonstrating the ability to induce membrane pore formation and lysis, effectively mimicking the membrane-disrupting mechanism of natural HDPs. This unique membranolytic action enabled D_G3_M_2_ to efficiently eliminate melanoma cells while sparing normal cells. In vivo, D_G3_M_2_ successfully inhibited tumor growth in a melanoma-bearing nude mice model with minimal systemic toxicity and no observable damage to vital organs, such as the liver and kidneys. Additionally, when compared to its activity against A549 lung cancer cells, D_G3_M_2_ showed superior anticancer effects against A375 melanoma cells, underscoring its potential for targeted melanoma therapy. Overall, these findings suggest that DMs, particularly D_G3_M_2_, hold significant promise as selective, biocompatible anticancer agents for melanoma treatment, warranting further investigation in preclinical and clinical studies.

## Data Availability

The original contributions presented in this study are included in the article/Supplementary Material. Further inquiries can be directed to the corresponding authors.

## Supplementary Materials

The following supporting information can be downloaded at: https://www.mdpi.com/article/10.3390/pharmaceutics17030361/s1, Figure S1: The chemical structure and schematic representation of DMs. Figure S2: Cytotoxicity of ε-polylysine, D_G1_, and D_G2_ as determined by CCK-8 assay; Figure S3: Inhibition rate of A375 and HaCaT cells after treatment with DMs at different concentrations and incubation times. Figure S4: The killing kinetic assay of DMs against A375 (a) and HaCaT cells (b) as determined by live/dead staining assay.

## References

[1] Arnold M., Singh D., Laversanne M., Vignat J., Vaccarella S., Meheus F., Cust A.E., de Vries E., Whiteman D.C., Bray F. (2022). Global Burden of Cutaneous Melanoma in 2020 and Projections to 2040. JAMA Dermatol..

[2] Ferlay J., Colombet M., Soerjomataram I., Parkin D.M., Piñeros M., Znaor A., Bray F. (2021). Cancer statistics for the year 2020: An overview. Int. J. Cancer.

[3] Lin L.F., Li Z.Y., Yan L., Liu Y.L., Yang H.J., Li H. (2021). Global, regional, and national cancer incidence and death for 29 cancer groups in 2019 and trends analysis of the global cancer burden, 1990–2019. J. Hematol. Oncol..

[4] Zhang Y.L., Hou J.B., Shi S.M., Du J., Liu Y.D., Huang P., Li Q., Liu L.C., Hu H.R., Ji Y.C. (2021). CSN6 promotes melanoma proliferation and metastasis by controlling the UBR5-mediated ubiquitination and degradation of CDK9. Cell Death Dis..

[5] Shirley C.A., Chhabra G., Amiri D., Chang H., Ahmad N. (2024). Immune escape and metastasis mechanisms in melanoma: Breaking down the dichotomy. Front. Immunol..

[6] Leonardi G.C., Falzone L., Salemi R., Zanghì A., Spandidos D.A., McCubrey J.A., Candido S., Libra M. (2018). Cutaneous melanoma: From pathogenesis to therapy (Review). Int. J. Oncol..

[7] Curti B.D., Faries M.B. (2021). Recent Advances in the Treatment of Melanoma. N. Engl. J. Med..

[8] Rui R., Zhou L.Q., He S.M. (2023). Cancer immunotherapies: Advances and bottlenecks. Front. Immunol..

[9] Pham J.P., Joshua A.M., da Silva I.P., Dummer R., Goldinger S.M. (2023). Chemotherapy in Cutaneous Melanoma: Is There Still a Role?. Curr. Oncol. Rep..

[10] Liu Q., Das M., Liu Y., Huang L. (2018). Targeted drug delivery to melanoma. Adv. Drug Deliv. Rev..

[11] Mundra V., Li W., Mahato R.I. (2015). Nanoparticle-mediated drug delivery for treating melanoma. Nanomedicine.

[12] Lu F.T., Zhu Y.K., Zhang G.D., Liu Z.P. (2022). Renovation as innovation: Repurposing human antibacterial peptide LL-37 for cancer therapy. Front. Pharmacol..

[13] Riedl S., Zweytick D., Lohner K. (2011). Membrane-active host defense peptides—Challenges and perspectives for the development of novel anticancer drugs. Chem. Phys. Lipids.

[14] Kardani K., Bolhassani A. (2021). Antimicrobial/anticancer peptides: Bioactive molecules and therapeutic agents. Immunotherapy.

[15] Szlasa W., Zendran I., Zalesinska A., Tarek M., Kulbacka J. (2020). Lipid composition of the cancer cell membrane. J. Bioenerg. Biomembr..

[16] Tornesello A.L., Borrelli A., Buonaguro L., Buonaguro F.M., Tornesello M.L. (2020). Antimicrobial Peptides as Anticancer Agents: Functional Properties and Biological Activities. Molecules.

[17] Quemé-Peña M., Ricci M., Juhász T., Horváti K., Bosze S., Biri-Kovács B., Szeder B., Zsila F., Beke-Somfai T. (2021). Old Polyanionic Drug Suramin Suppresses Detrimental Cytotoxicity of the Host Defense Peptide LL-37. ACS Pharmacol. Transl. Sci..

[18] Mookherjee N., Anderson M.A., Haagsman H.P., Davidson D.J. (2020). Antimicrobial host defence peptides: Functions and clinical potential. Nat. Rev. Drug Discov..

[19] Ergene C., Yasuhara K., Palermo E.F. (2018). Biomimetic antimicrobial polymers: Recent advances in molecular design. Polym. Chem..

[20] Kalelkar P.P., Riddick M., García A.J. (2022). Biomaterial-based antimicrobial therapies for the treatment of bacterial infections. Nat. Rev. Mater..

[21] Dias A.P., Santos S.D., da Silva J.V., Parise R., Ferreira E.I., El Seoud O., Giarolla J. (2020). Dendrimers in the context of nanomedicine. Int. J. Pharm..

[22] Chauhan A.S. (2018). Dendrimers for Drug Delivery. Molecules.

[23] Wang J., Li B.X., Qiu L., Qiao X., Yang H. (2022). Dendrimer-based drug delivery systems: History, challenges, and latest developments. J. Biol. Eng..

[24] Qian Y.S., Yang D.J., Zhu J.M., Huang S.T., Chen S.J., Zeng J., Xu J., He J., Zhou C.C. (2024). Mimics of Host Defense Peptides Derived from Dendronized Polylysines for Antibacterial and Anticancer Therapy. ACS Macro Lett..

[25] Burton K.A., Ashack K.A., Khachemoune A. (2016). Cutaneous Squamous Cell Carcinoma: A Review of High-Risk and Metastatic Disease. Am. J. Clin. Dermatol..

[26] Seidel J.A., Otsuka A., Kabashima K. (2018). Anti-PD-1 and Anti-CTLA-4 Therapies in Cancer: Mechanisms of Action, efficacy, and Limitations. Front. Oncol..

[27] Janaszewska A., Lazniewska J., Trzepinski P., Marcinkowska M., Klajnert-Maculewicz B. (2019). Cytotoxicity of Dendrimers. Biomolecules.

[28] Chis A.A., Dobrea C., Morgovan C., Arseniu A.M., Rus L.L., Butuca A., Juncan A.M., Totan M., Vonica-Tincu A.L., Cormos G. (2020). Applications and Limitations of Dendrimers in Biomedicine. Molecules.

[29] Tian Y.H., Li S.P., Song J., Ji T.J., Zhu M.T., Anderson G.J., Wei J.Y., Nie G.J. (2014). A doxorubicin delivery platform using engineered natural membrane vesicle exosomes for targeted tumor therapy. Biomaterials.

[30] Schweizer F. (2009). Cationic amphiphilic peptides with cancer-selective toxicity. Eur. J. Pharmacol..

[31] Gajski G., Garaj-Vrhovac V. (2013). Melittin: A lytic peptide with anticancer properties. Environ. Toxicol. Pharmacol..

[32] Zhou X.Y., He J., Zhou C.C. (2019). Strategies from nature: Polycaprolactone-based mimetic antimicrobial peptide block copolymers with low cytotoxicity and excellent antibacterial efficiency. Polym. Chem..

[33] Shao N., Yuan L., Ma P.C., Zhou M., Xiao X.M., Cong Z.H., Wu Y.M., Xiao G.H., Fei J., Liu R.H. (2022). Heterochiral β-Peptide Polymers Combating Multidrug-Resistant Cancers Effectively without Inducing Drug Resistance. J. Am. Chem. Soc..

[34] Kumar P., Kizhakkedathu J.N., Straus S.K. (2018). Antimicrobial Peptides: Diversity, Mechanism of Action and Strategies to Improve the Activity and Biocompatibility In Vivo. Biomolecules.

