# Supplementary material for: Dendrimer-Derived Mimics of Host Defense Peptides Selectively Disrupt Cancer Cell Membranes for Melanoma Therapy
Source: Pharmaceutics. 2025 Mar 12;17(3):361. doi: 10.3390/pharmaceutics17030361 (PMC11946345; doi:10.3390/pharmaceutics17030361)
Supplement: Supplementary file 1 [file pharmaceutics-17-00361-s001.zip › pharmaceutics-3474371-supplementary.pdf]

Supporting Information for

Dendrimer-derived Mimics of Host Defense Peptides Disrupt-ing Cancer Cell Membranes Selectively for Melanoma Therapy

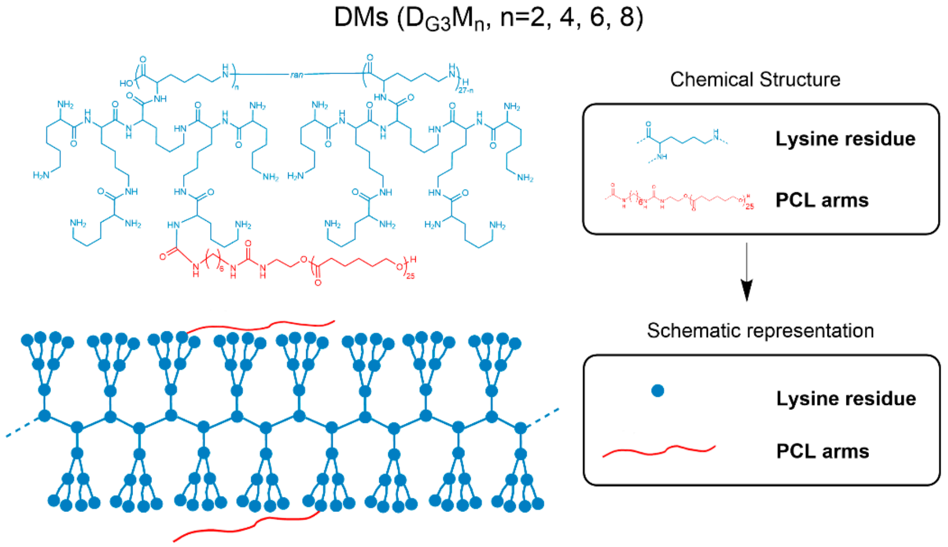

Figure S1. The chemical structure and schematic representation of DMs.

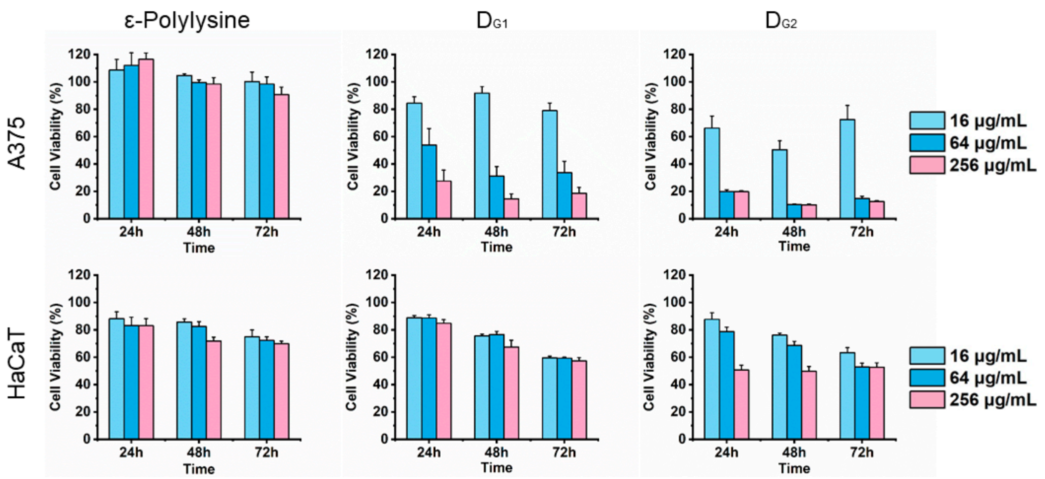

Figure S2. Cytotoxicity of ε-polylysine, D_{G1}, and D_{G2} as determined by CCK-8 assay.

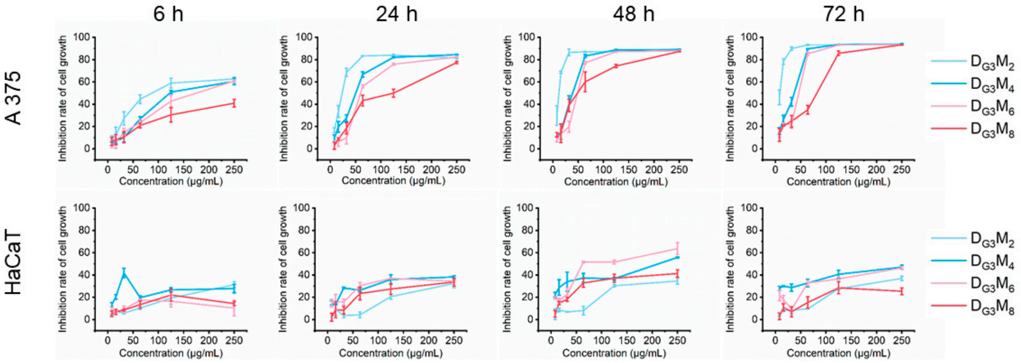

Figure S3. Inhibition rate of A375 and HaCaT cells after treatment with DMs at different concentrations and incubation times.

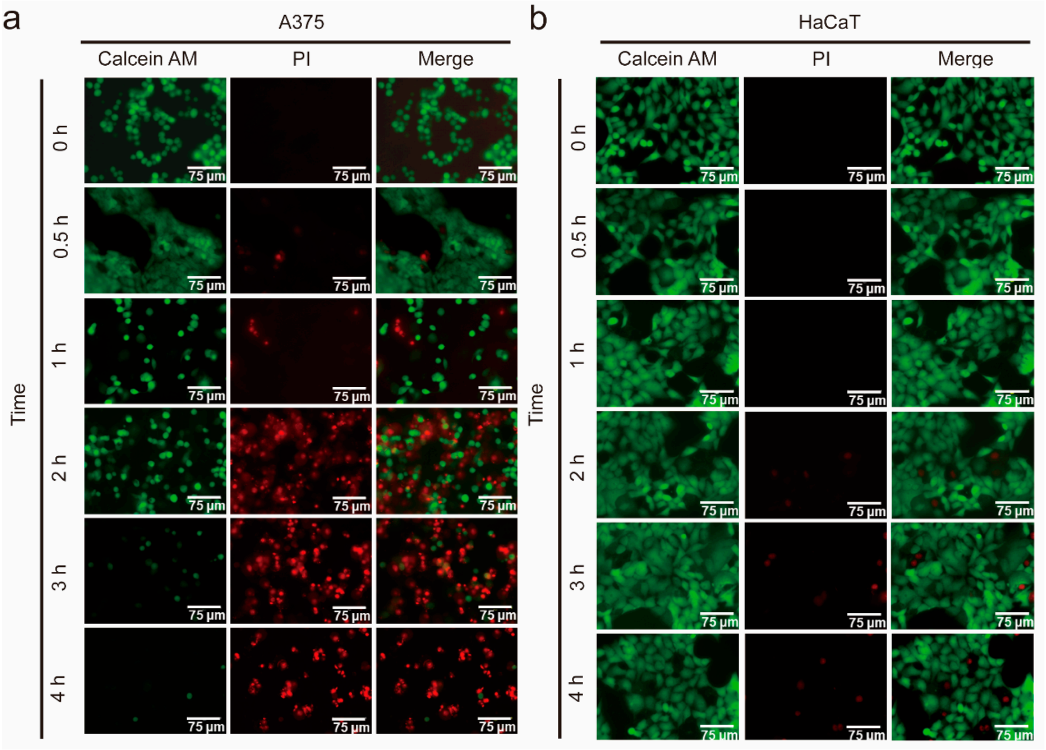

Figure S4. The killing kinetic assay of DMs against A375 (a) and HaCaT cells (b) as determined by live/dead staining assay.
